# Effective weight loss after treatment with a glucagon-like peptide-1 receptor agonist in a morbidly obese and diabetic patient before bariatric surgery: a case report

**DOI:** 10.1186/1752-1947-8-304

**Published:** 2014-09-11

**Authors:** Jui-Hung Chen, Wen-Hao Tang, Chien-Hsing Lee

**Affiliations:** 1 Department of Internal Medicine, Tri-Service General Hospital Songshan Branch, National Defense Medical Center, No.131, Jiankang Rd, Songshan District, Taipei City 10581, Taiwan; 2 Division of Endocrinology and Metabolism, Department of Internal Medicine, Tri-Service General Hospital, National Defense Medical Center, No. 325, Sec. 2, ChengGong Rd, NeiHu District, Taipei City 114, Taiwan

**Keywords:** Bariatric surgery, Diabetes, GLP-1 receptor agonist, Morbid obesity

## Abstract

**Introduction:**

Glucagon-like peptide-1 receptor agonists, a new class of anti-diabetic drugs, are widely used in the treatment of type 2 diabetes. However, the effect of glucagon-like peptide-1 receptor agonists on the treatment of preoperative weight loss in obese type 2 diabetic patients has not been reported.

**Case presentation:**

A 38-year-old Taiwanese woman presented to our hospital with morbid obesity and type 2 diabetes mellitus. Bariatric surgery was recommended by a general surgery specialist. Weight loss before surgery was recommended to reduce the frequency of surgical complications. In addition to diet control with lifestyle modifications, pharmacological treatment with metformin and glucagon-like peptide-1 receptor agonists was administered. Fourteen months of treatment reduced her hemoglobin A1c level from 7.4 to 5.5% and reduced her body weight by 21.2kg.

**Conclusions:**

One year of diet control with lifestyle modifications and pharmacological treatment with glucagon-like peptide-1 receptor agonists and metformin markedly decreased hemoglobin A1c levels and resulted in effective and substantial weight loss in a morbidly obese patient with dysregulated diabetes during the preoperative period.

## Introduction

Bariatric or metabolic surgery can rapidly improve glycemic control and cardiovascular risk factors in severely obese patients with type 2 diabetes [1-3]. Growing evidence has revealed that preoperative weight loss before surgery plays a crucial role in the preparation of morbidly obese patients during bariatric surgery [4-6]. Preoperative weight loss may be associated with a reduced risk of surgical complications in bariatric surgery. However, the optimal method of preoperative weight loss remains controversial; these methods include a low calorie or very low calorie diet or the placement of an intragastric balloon. Recently, glucagon-like peptide-1 (GLP-1) receptor agonists, such as exenatide and liraglutide, can efficiently reduce blood glucose levels, enhance satiety and reduce energy intake through centrally mediated mechanisms [7,8]. These GLP-1 receptor agonists are now used increasingly in the treatment of obesity-related type 2 diabetes, but the effects of these drugs in preoperative weight loss are not known. We report the case of a patient with type 2 diabetes and severe obesity who had an excellent therapeutic response to diet control with lifestyle modifications and GLP-1 receptor agonists, which included significant improvements in weight and glycemic control.

## Case presentation

A 38-year-old Taiwanese woman, with a past history (more than five years) of hypertension, presented to our outpatient clinic in May 2012 with several months of deteriorated shortness of breath and dyspnea upon exertion. She weighed 170kg with a body mass index (BMI) of 69.9kg/m^2^, and her fasting glucose level was 132mg/dl. Under the diagnosis of morbid obesity and suspected diabetes mellitus, she was admitted to our ward. She reported no history of gestational diabetes mellitus, with a baby birth weight of approximately 3600gm. However, her mother has a family history of type 2 diabetes mellitus.

**Figure 1:**
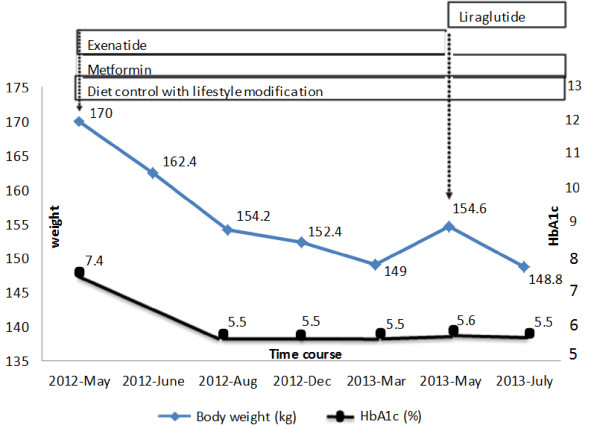
**Course of treatment graph showing the corresponding effect on weight and HbA1c level.** The duration of treatment with diet control, lifestyle modifications and pharmacological agents in our patient is represented by the length of each box relative to the time period on the x-axis. Changes in HbA1c are shown by the black circle line plotted against the y-axis (right), and changes in weight are shown by the blue rhombus line plotted against the y-axis (left). Significant improvement in glycemic control and sustained weight loss after metformin and exenatide is shown.

Upon admission, her body temperature was 36.6°C, her pulse rate was 101/min, her respiratory rate was 20/min, and her blood pressure was 146/89mmHg. Physical examinations revealed hyperpigmentation over the neck and axillary regions. During hospitalization, her hemoglobin A1C (HbA1c) was 7.4%. Her other endocrinologic blood examinations, such as free T4, human growth hormone, cortisol, 24-hours urine free cortisol, adrenocorticotropic hormone (ACTH), follicle-stimulating hormone (FSH), luteinizing hormone (LH), prolactin, estradiol, progesterone, testosterone and dehydroepiandrosterone sulfate (DHEA-S) were within normal limits, however, her thyroid-stimulating hormone (TSH) was mildly elevated (5.81μIU/ml).Bariatric surgery was recommended by a general surgery (GS) specialist, however, she was warned about the high risk of complications associated with the surgery. Diet control with lifestyle modifications and pharmacological treatment for weight loss before surgery were recommended to reduce the chance of surgical complications. We consulted dietitians and a high fiber diabetic full diet with a calorie restriction of 1800kcal/day was recommended. In addition to diet control with lifestyle modifications, pharmacological treatment with metformin (1g/day) and a GLP-1 receptor agonist (exenatide 20mg/day via subcutaneous injection) were administered. Following the initiation of metformin and exenatide, she experienced weight loss, which continued throughout the therapy until May 2013. She reported poor compliance of exenatide at a twice daily usage, therefore we replaced exenatide with liraglutide (1.2mg/day via subcutaneous injection). Fourteen months of treatment reduced her HbA1c level to 5.5% and reduced her body weight by 21.2kg (Figure 1). She reported no problems with gastrointestinal distress or hypoglycemia and noted a slight decrease in appetite. She is currently under regular GS outpatient clinic follow-up and bariatric surgery has been arranged.

## Discussion

We reported that one year of diet control with lifestyle modifications and GLP-1 receptor agonist treatment markedly decreased HbA1c levels and resulted in a substantial weight loss in a severely obese patient with dysregulated diabetes. To the best of our knowledge, this is the first clinical evidence supporting the use of GLP-1 receptor agonists on the treatment of preoperative weight loss in obese type 2 diabetic patients.

Bariatric surgery may be considered for adults with a BMI of ≧35kg/m^2^ and type 2 diabetes, especially if the diabetes or associated comorbidities are difficult to control with lifestyle modifications and pharmacological therapy [9]. Recently, the topic of preoperative weight loss before bariatric surgery has garnered increasing attention. Several studies have demonstrated that preoperative weight loss resulted in decreased operative time, perioperative complications and greater total postoperative weight loss. Livhits *et al.*[5] declared that preoperative weight loss immediately before bariatric surgery appears to improve the total postoperative weight loss and decrease the operative time. A modest decrease of 10% of excess body weight will improve a patient’s respiratory mechanics and decrease both cardiovascular and thromboembolic risk factors, chronic pro-inflammatory status and serum glucose concentrations [10,11]. In addition to the potential to improve global patient risk factors, preoperative weight loss also decreases intra-abdominal fat stores. The proposed mechanism involves the shrinkage of visceral adiposity and intrahepatic fat [12].

The methods of preoperative weight loss differed among reported studies but generally included a nutritional component under the direction of a registered dietician and an exercise plan. Several studies demonstrated a positive effect of preoperative weight loss with a low calorie liquid diet, and most studies included dietary logs and nutritional education. A very low energy diet instituted before bariatric surgery has been shown to decrease the liver volume and visceral and subcutaneous adipose tissue in proportion to the reduction in body weight [6]. A successful preoperative weight loss program will likely contain both exercise and a nutritional component, with the latter providing specific dietary instructions and specialized counseling.

With the exception of diet control with lifestyle modifications, many review articles reaffirm the weight benefits of metformin alone or in combination with other anti-diabetic agents that are not associated with weight gain for the treatment of diabetes and body weight loss [9]. GLP-1 receptor agonists, a new class of anti-diabetic drugs, are widely used in the treatment of type 2 diabetes [7]. GLP-1 receptor agonists stimulate glucose-induced insulin secretion, inhibit glucagon secretion and reduce gastrointestinal motility, which reduces appetite and food intake [8]. As shown in previous studies, exogenously administered GLP-1 receptor agonists have a therapeutic role in restoring beneficial effects to decrease HbA1c by approximately 1% and to promote weight loss of 2 to 3kg over six months [13]. Randomized placebo-controlled trials using exenatide in patients with type 2 diabetes have shown a mean weight reduction of between 2 and 3kg at 30 weeks, and nearly 5kg (5% excess weight loss (%EWL) from baseline) in open-label extension studies of up to two years [14,15]. Our patient had a 21.2kg (12.4%EWL) weight reduction from baseline using diet control with lifestyle modifications, metformin and GLP-1 receptor agonists, which was a better result than the published clinical trials, and this benefit was sustained over the following year. Our treatment also efficiently reduced blood glucose levels and no episode of hypoglycemia has occurred since the treatment.

## Conclusions

As mentioned above, preoperative weight loss appears to be associated with greater weight loss postoperatively and is associated with fewer complications after gastric bypass surgery. Different methods, such as a low or very low calorie diet or an intragastric balloon, were used for preoperative weight loss just before bariatric surgery. In this study, diet control with lifestyle modifications and pharmacologic therapy with GLP-1 receptor agonists resulted in clinically relevant beneficial effects on body weight loss and blood sugar control before bariatric surgery. Therefore, in addition to diet control and lifestyle modifications, GLP-1 receptor agonists would appear to be an important therapeutic option in a morbidly obese patient with type 2 diabetes before undergoing bariatric surgery. Randomized controlled clinical trials of GLP-1 receptor agonists as preoperative weight-reducing agents in severely obese patients with type 2 diabetes are warranted.

## Consent

Written informed consent was obtained from the patient for publication of this case report and any accompanying images. A copy of the written consent is available for review by the Editor-in-Chief of this journal.

## Abbreviations

BMI: Body mass index; EWL: Excess weight loss; GLP-1 receptor agonists: Glucagon-like peptide-1 receptor agonists; GS specialist: General surgery specialist.

## Competing interests

The authors declare that they have no competing interests.

## Authors’ contributions

JHC wrote the manuscript draft and made corrections to manuscript in light of reviewers’ comments. WHT followed up this patient as an outpatient and contributed to the discussion. CHL performed critical revision of the manuscript and supervised the study. All authors read and approved the final manuscript.
